# Reputation, a universal currency for human social interactions

**DOI:** 10.1098/rstb.2015.0100

**Published:** 2016-02-05

**Authors:** Manfred Milinski

**Affiliations:** Department of Evolutionary Ecology, Max Planck Institute for Evolutionary Biology, Plön, Germany

**Keywords:** reputation, social dilemma, indirect reciprocity, updating rule, gossip

## Abstract

Decision rules of reciprocity include ‘I help those who helped me’ (direct reciprocity) and ‘I help those who have helped others’ (indirect reciprocity), i.e. I help those who have a reputation to care for others. A person's reputation is a score that members of a social group update whenever they see the person interacting or hear at best multiple gossip about the person's social interactions. Reputation is the current standing the person has gained from previous investments or refusal of investments in helping others. Is he a good guy, can I trust him or should I better avoid him as a social partner? A good reputation pays off by attracting help from others, even from strangers or members from another group, if the recipient's reputation is known. Any costly investment in others, i.e. direct help, donations to charity, investment in averting climate change, etc. increases a person's reputation. I shall argue and illustrate with examples that a person's known reputation functions like money that can be used whenever the person needs help. Whenever possible I will present tests of predictions of evolutionary theory, i.e. fitness maximizing strategies, mostly by economic experiments with humans.

## 1. Introduction

For direct reciprocity you need a face, for indirect reciprocity you need a name (David Haig, Harvard University).

Cooperation implies costs and benefits for either partner. If investments do not pay off immediately, because the partner invests later, there is the risk of being exploited [1]. No guarantee exists that the partner will reciprocate. The investor has to trust the partner. The experimental paradigm to study this scenario is the trust game (e.g. [2]). There are two players, the sender and the receiver. The sender is given, e.g. €10 that he can either retain or send to the receiver who receives it tripled: €30. The receiver can retain the money or send any amount back to the sender. The fair share would be to send back €15. Should the sender risk losing their €10 hoping to receive €15? Any information about the partner's trustworthiness would reduce the risk of deceit. They may observe the receiver's potential smile. A ‘Duchenne smile’ is costly to produce [3] and thus assumed to be an honest signal of trustworthiness. It has been predicted [4] and shown that receivers produce it with high probability when they have decided to send money back, but only if the stakes are high in the trust game. When only little money can be gained they do not smile ‘Duchenne’ [5]. Another way to learn whether a partner can be trusted is observing ‘eavesdropping’ [6,7], a potential partner's interactions with somebody else. It is not only humans who profit from eavesdropping to decide whether to team up with somebody [8], song sparrows have also been shown to distrust aggressive neighbours based on eavesdropping [9]. The best way to judge the trustworthiness of a partner would be to know the proportion of all their previous social interactions in which they reciprocated help or invested in others, i.e. their reputation. I will show that reputation can be gained by any kind of altruistic investment, e.g. helping group mates or donating money for refugees. All adds up in a single currency—reputation. Therefore, I call it a universal currency that can be used in any other social interaction. Some theoretical studies of the evolution of cooperation assume that a person's reputation is public knowledge (e.g. [10]), known either from direct observation of social interactions or from gossip. Indeed, especially multiple gossip about a person's social behaviour can rather reliably spread a person's reputation [11]. Reputation gained via reciprocating also helps in the trust game; the senders' decisions were strongly influenced by the gossip that was based on reciprocating behaviour [11]. This shows the strong relationship between reciprocity, trust and reputation as described by Ostrom [12]. Reputation is not always positive: it is negative when a person has mostly refused help. As Trivers [13] points out, a social trait affects at least one individual other than the possessor of the trait with the effect that the actor confers a benefit but suffers a cost, the actor gains but inflicts a cost, both parties gain, or both partners suffer. Social behaviour can thus be rather unsocial conferring a negative reputation to the actor. I will review experimental studies of the role of reputation in human social interactions, preferentially when studies tested predictions of evolutionary theory: which strategy do we expect to have evolved maximizing the actor's fitness? I concentrate on studies with humans, because they are more numerous than studies in non-human animals. This does not mean that any kind of reputation management is rare in animals although gossip might be difficult. It is much more difficult and laborious to study animals [14]; thus researchers such as myself study reciprocity mostly in humans.

## 2. Direct reciprocity

If I help you and later you help me, we have reciprocated help. With the cost of helping being low and the benefit from receiving help being high, we both gain a net benefit. Trivers [1] listed further conditions for ‘direct reciprocity’ to work, such as the two individuals need to have a high probability to meet again and they must trust each other: there is the risk of cheating when it comes to reciprocating help. Trivers already saw that the Prisoner's Dilemma (see [15] for review) describes the scenario of the two reciprocators. If both cooperate they both earn reasonably well, say €3 each. If one cooperates and the other defects, the defector earns €5 and the sucker 0. If both defect, they receive €1 each. Whatever the other one does, I earn more when I defect, hence both defect and earn €1 each instead of €3 each had they both cooperated, hence the dilemma. If, however, the game is played repeatedly with no known last round, numerous strategies of playing C or D are possible, preventing an analytical solution for finding the best strategy. Axelrod [16] invited the brightest minds to provide their favourite strategy for a computer tournament. Tit for tat (tft), a cooperative, retaliating and forgiving strategy was the champion [17]—cooperate on the first move and then copy your partner's previous move. Some studies found tft-like strategies both in animals (e.g. [18–22] and others) and humans (e.g. [23]). Further champions were similar, such as generous tft [24] and ‘win-stay, lose-shift’ [25,26], and kept the championship for almost 20 years until Press & Dyson [27] described a class of ‘zero-determinant’ strategies including extortionate strategies that beat any adaptive strategy including tft, etc. In the iterated Prisoner's Dilemma, the opponent can only gain most by maximal cooperation; however, their extortionate partner always gains more with the opponent's increasing cooperation. Humans are easily exploited by extortioners, yet not fully, because they eventually cease cooperating and lose money, providing the extortioner with a greater loss of expected gain in an experiment [28]. The bad reputation of being an extortioner might spread quickly, probably preventing the strategy from becoming frequent. All the champion strategies rely on a short memory of up to two previous interactions. They might be beaten by a strategy that learns over many interactions how trustworthy a partner is. Their reputation as a reciprocator may spread through multiple gossip [11,29] and become public knowledge in a community.

## 3. Indirect reciprocity

In indirect reciprocity [30,31], a prediction from the bible is verified—‘help and you shall receive’. Indirect reciprocity has two steps:

(1) A observes that B helps C.
(2) A helps B.

Nowak & Sigmund [32,33] showed by both computer simulations and an analytical model that cooperation through indirect reciprocity can evolve if everybody has an ‘image score’ that is increased by one point after each act of helping and is decreased by one point after each act of refused help. A helps B only if they have a positive image score, i.e. reputation. If a person has helped more often than they have refused help, they can expect help from others.

Wedekind & Milinski [34] tested whether Swiss students would give money to others with a known image score even if they were told that direct reciprocity was excluded. If they did give money, would they preferentially help those who had a positive image score? Eight groups with 10 students each were tested. Each subject was anonymized with a pseudonym. The benefit of giving was 4 Swiss francs for the receiver, whereas the cost for the donor was 2 Swiss francs taken from their account. Each subject played once in each of six rounds both as ‘donor’ and as ‘receiver’. Each pair of players was randomly chosen, and interacted in public under their pseudonyms. Everybody could see all of the previous donor choices, ‘yes’ and ‘no’, of the receiver, when the donor was asked ‘would you give 2 Swiss francs to player x, yes or no?’ After the game, everybody received their money in a way that did not disclose the players' identity.

**Figure 1.**
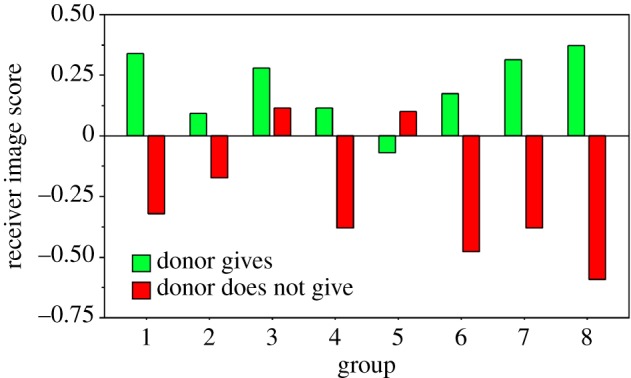
Green columns show the average image score of all the receivers who received money and red columns show the average image score of all the receivers who did not receive anything in each of the eight groups. Data are shown as deviations from the means per group and round to correct for group and round effects (adapted from [34]).

The receivers' reputation had a significant effect on the donors' decisions whether to give money: the image score of receivers who were given money was on average higher than the score of those who got nothing in all but one of the eight groups (figure 1). Those who gave rarely supported only players who had a very high image score. Other experimental studies found similar results [35–40]. Thus, human subjects who have been helpful in the past are likely to receive help from others through indirect reciprocity. A positive reputation pays off in indirect reciprocity.

If individuals repeatedly interact within small groups with different partners in a two person Prisoner's Dilemma, cooperation can emerge and also be maintained in the absence of cognitive capabilities. It is sufficient for an individual to base their decision of whether or not to cooperate on the outcome of their last encounter. Such generalized reciprocity, or upstream reciprocity, does not afford reputation mechanisms ([41]; see also [42,43] for upstream reciprocity). It is thus not a topic of this review. Here I review the role of reputation in social interactions.

Another mechanism that can enhance cooperation is reputation-based partner choice, or competitive altruism if there is competition for the attention of other altruists or competition for mates [44,45].

## 4. Reputation dynamics—standing or scoring

Other theorists [46] questioned the suitability of the scoring strategy and showed theoretically with a more complex population structure that Sugden's [47] ‘standing’ strategy is superior in establishing cooperation through indirect reciprocity. Using the image scoring strategy a player would correctly refuse to help an individual with a low score thereby reducing their own score. This player might suffer from not being helped thereafter. In Sugden's [47] standing model everybody starts in good standing, but loses good standing by failing to help a recipient in good standing. However, failing to help recipients who lack good standing does not damage the standing of a donor. It seems to be fair to refuse to help someone who lost their good standing without losing one's own good standing. Ohtsuki & Iwasa [48] did an in-depth analysis of these two and several other reputation updating rules for indirect reciprocity and found standing strategies to be more successful than scoring strategies in most cases. The updating rule defines the ‘reputation dynamics’ of a person [48].

**Figure 2.**
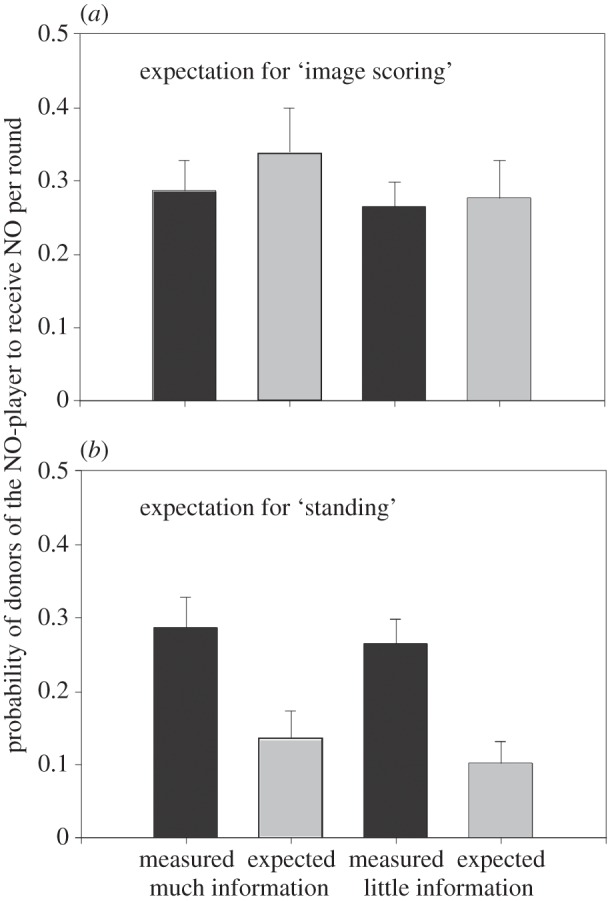
Strategies for reputation dynamics, image scoring and standing. Mean (+s.e.) probabilities of donors of NO-players receiving NO per round (during 16 rounds) of 12 groups with much information and 11 groups with little information of six subjects plus one NO-player each. (*a*) Expectation for image scoring: measured probabilities (filled bars) are compared with expected probabilities (grey bars). (*b*) Expectation for standing. The black bars are the same in (*a*) and (*b*) showing measured behaviour. The grey bars show the average values calculated for the respective decisions assuming (*a*) image scoring and (*b*) standing (adapted from [36]).

We should study which strategy human subjects actually adopt when they participate in indirect reciprocity games. Because the results of previous experimental studies are compatible with either strategy, Milinski *et al.* [36] addressed the preference for standing versus scoring in a specific experiment. Each group included a secretly instructed NO-player who always refused to help. Both standing and scoring would always refuse to give to such a player. The decisive question is, would *their* potential donors in turn penalize these players? Image scorers would penalize them, standing players would not, because not helping NO-players is justified. Figure 2 shows that these donors of the NO-players were penalized almost as often as predicted for scoring but much more often than expected from standing. Moreover, they compensated for NOs to the NO-player by fewer NOs to others, which would not make sense if they expected their co-players to follow a standing strategy. Providing the subjects with first plus second order information (‘much information’) compared with first-order information only (‘little information’) appeared to have no obvious effect on the donors' strategy (figure 2), probably because all players had directly observed all previous interactions. Bolton *et al.* [35] obtained similar results.

Why do humans not adopt the superior reputation updating strategy of standing? They use scoring, which is not among Ohtsuki and Iwasa's ‘leading eight’. They [48] assume that each player has a binary reputation, either good or bad, which is known publicly. This is a perfect world. In reality, people do not know about all interactions. Missing information can be filled by gossip to some extent [11]. If your potential receiver has decided ‘no’ in their last interaction as a donor, you need to know whether this ‘no’ was justified to determine whether they are now in good standing and you will help them according to a standing strategy. If they had responded with their ‘no’ to a ‘no’ of their previous receiver that they had decided as a donor, you need to go further back to previous interactions until you know whether the last ‘no’ you encounter was justified or not, to determine whether your current receiver's ‘no’ is justified and they are in good standing. If information about only one interaction is missing, you cannot decide how to respond to your present receiver. This is different with an image scoring strategy. A receiver's image score is the net sum of all ‘yes' and ‘no’ they have decided in the past. A rather cooperative person will have a positive image score of, say, +15. It does not matter if information about a few interactions is missing, their image score will still be positive and you help them if you use a scoring reputation updating strategy. Standing strategies might have failed too often in the past, so that humans today prefer the less precise but applicable reputation updating strategy of scoring. New theoretical approaches have approved the above reasoning formally and have re-established the validity of the scoring strategy [49,50], but see [51] for a different solution.

## 5. Reputation helps solving the tragedy of the commons

If the Prisoner's Dilemma game is played with more than two players, it is a public goods game, i.e. the experimental paradigm to study a problem that Garrett Hardin [52,53] called ‘the tragedy of the commons'—‘Whenever people have free access to a public resource, the resource will be overused and collapse’. Examples proving Hardin right are plentiful: the classic ones are overexploiting fish stock and unlimited use of fossil energy that destroys the global climate through increasing CO_2_ emissions, followed by a steady increase of the global temperature (IPCC report [54]). The public goods game [55] is used to experimentally test hypotheses for solving the tragedy of the commons.

For a typical public goods game four subjects are given an endowment from which they can each invest anonymously either €1 or nothing into a public account in each round of the game. After each round the money in the account is doubled and redistributed to all players irrespective of whether they have contributed. If all invest, each has a net gain of €1. If three players invest, each investor gains €0.50; however, the defector gains €1.50. Defectors always gain more than cooperators. With three defectors and one cooperator, each defector gains €0.50; the cooperator, however, loses €0.50. This shows that you gain less than you have invested. The gain maximizing strategy in a public goods game is to never invest anything. You hope that others are irrational and invest. Usually public goods games start with unpredicted cooperation, which quickly declines during consecutive rounds (e.g. [55]). The public goods game can be combined with the opportunity either to punish or to reward specific co-players. If, for example, each public goods round is followed by a round where each player can be punished, e.g. loses €3 and the punisher pays €1, defectors are punished preferentially and, as a consequence, contributions to the public good are maintained at a high level—the tragedy of the commons is resolved (e.g. [56–59]). However, due to the cost of punishing and of being punished there is not usually a net gain from increased cooperation ([60], but see [61]).

When players can choose between joining a public goods game with and without the punishment opportunity repeatedly for 20 rounds, they increasingly choose having the punishment option [62]. However, in the initial rounds the majority opt against having the punishment possibility. Having a single initial vote, the majority of subjects typically decide for reward instead of punishment [63]. Is rewarding a viable alternative to sustain high contributions in public goods games? Considerable evidence has been collected showing that it is not. In direct comparisons the possibility of directly rewarding co-players does not yield higher cooperation levels than punishment [63,64] and among experienced players punishment is even more successful in fostering cooperation than rewarding ([65,66]; see [67] for a review). Interestingly, subjects gain reputation by rewarding but not by punishing others. Even ‘justified’ punishment of defectors is neutral with regard to reputation ([8]; see also [68]).

**Figure 3.**
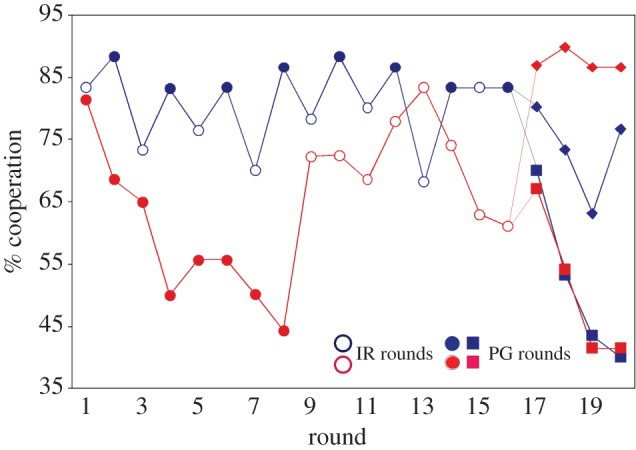
Percentage of cooperation (‘yes’) per group of six subjects in each round of the public goods game (filled symbols) and in each round of the indirect reciprocity game (open symbols). In one treatment, the groups alternated between rounds of indirect reciprocity and rounds of public goods game until round 16 (blue); in the other treatment, groups started with eight consecutive rounds of the public goods game and continued with eight rounds of the indirect reciprocity game (red); in rounds 17–20, groups of both treatments played the public goods game, which was either announced (squares) or not announced (diamonds) (adapted from [69]).

A less costly way than punishment to induce cooperation in the public goods game is provided by combining the public goods game with a game in which a good reputation is necessary for gaining money [69,70]. If rounds of the public goods game are alternated with rounds of the indirect reciprocity game and players have the same pseudonym in both games, the public good is maintained at the high starting level [69] (figure 3). Players who did not contribute to the public good in one round were less often rewarded in the next indirect reciprocity round than those that had contributed. Thus a good reputation gained in the public goods game is rewarded in the indirect reciprocity game and defectors do not risk losing their reputation. When this risk is removed by telling players after round 16 that only rounds of the public goods game will follow, contributions to the public good collapsed (figure 3). Thus, the pending risk of rounds where reputation matters had enforced cooperation. A theoretical analysis later showed that the reputation effect is predicted to be stable ([70]; see also [71,72]).

## 6. Strategic investment in reputation

The previous results suggest that humans invest in cooperation only if it will pay-off because their reputation is a valuable currency in the future. However, if the subject knows that they will not be recognizable as the person that did not cooperate in a previous game, will they cooperate less than if they know they will be recognized? Do humans invest strategically in reputation when they know that their costly investment will pay off in inevitable future interactions [73,74], and do they stop investing when this is not the case?

Semmann *et al.* [39] again alternated rounds of the public goods and the indirect reciprocity game, but this time allowed for reputation transfer from the public goods to the indirect reciprocity game only in some rounds but blocked this transfer in other rounds. The subjects were each provided with two different pseudonyms. One name, the non-transferable name (NT), was used only in some public goods rounds, whereas the other name, the transferable name (T), was used in rounds of both games. In each round of the public goods game the subjects knew whether they played with their transferable or non-transferable name. Would they invest less in the public pool when playing with the non-transferable name?

**Figure 4.**
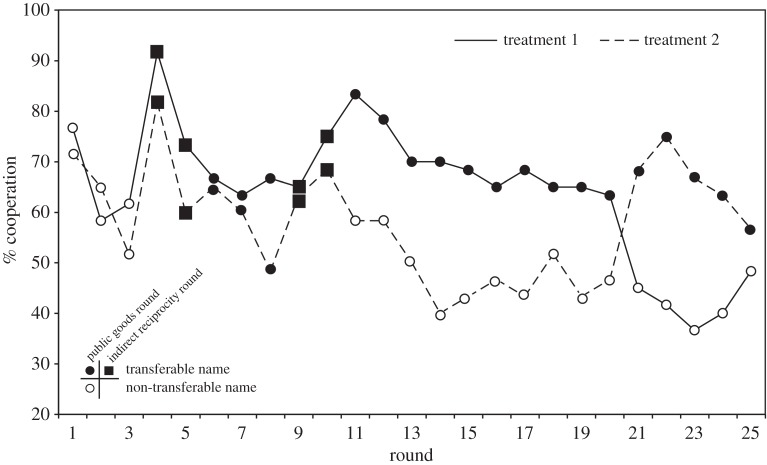
For the public goods (PG) rounds (circles) and indirect reciprocity (IR) rounds (squares), the group mean ‘yes’ per round for both treatments are shown. In treatment 1 (black), the groups played PG rounds, from round 11 to round 20 with their transferable name (T) (filled symbols) and from round 21 to 25 with their non-transferable name (NT) (open symbols). In treatment 2 (grey), the groups played PG rounds, from round 11 to round 20 with their non-transferable name and from round 21 to 25 with their transferable name. The period from round 1 to 10 was identical in both treatments (three PG rounds played with the non-transferable name, two IR rounds with the transferable name, three PG rounds with the transferable name and two IR rounds with the transferable name) (adapted from [39]).

After some training rounds, 10 groups with six subjects each played 10 consecutive rounds of the public goods game with their transferable names followed by five rounds with their non-transferable names. To control for sequence effects, 10 other groups played their first 10 consecutive rounds of public goods with their non-transferable names followed by five rounds with their transferable names. Figure 4 shows that the level of cooperation was much higher during the rounds with the transferable name. Hence, the subjects were either selfish or cooperative in the public goods game conditional on whether the player knew whether their decision would be known in another social game. The knowledge of being recognized as the same individual in both games motivated players to invest in their reputation and thereby sustain the public resource. Also in non-human animals, strategic reputation building seems possible. Bshary [75] suggested that tactical deception in cleaner fish should occur if it pays to alter the optimal behaviour in a situation to induce responses in bystanders (clients). Studies on ‘eavesdropping’ in animals (e.g. [9]) are suggestive of indirect reciprocity.

## 7. Eyes as signal of being observed

How do humans assess whether their actions are observed? Only subtle cues of being watched, such as two stylized eye-like shapes on the desktop background, suffice to change behaviour from selfish to social [76]. A photo showing a pair of eyes attached to a cafeteria honesty box significantly raises the donated amount compared with an attached flower symbol; the eyes were most effective when looking directly at the observer ([77]; see also [78]). Being just ink on paper, the signal obviously elicits unconscious hard-wired reactions. Electrophysiological approaches have been used in humans to determine responses to eye gaze. Event-related brain potential (ERP) responses recorded from the lateral temporal scalp of normal subjects showed larger responses to isolated eyes compared to full faces [79]. Neuro-imaging studies in humans have also highlighted a role for the superior temporal sulcus (STS) and amygdala in gaze processing; the STS is likely to be essential for recognizing the eyes, head and body as stimuli used in social communication, whereas the amygdala is likely to be essential for attaching socio-emotional significance to these stimuli [80]. The neurophysiological evidence suggests that to care about our reputation seems to be a ‘hardwired’ prerequisite of our social life (see review [81]). Also prosociality in infants and children can be seen as an example of evolved (gene programmed) cooperation, e.g. [82].

## 8. Reputation is valuable within and outside one's own social group

If players reward cooperative behaviour in the public goods game of their own group members and refuse to reward uncooperative behaviour, the incentive for donating in public goods games could be direct reciprocity, not necessarily reputation building. The interaction with indirect reciprocity should therefore sustain cooperation in public goods games only when the same players play in both games. We would expect that cooperative players from another public goods group should not be rewarded in indirect reciprocity games.

**Figure 5.**
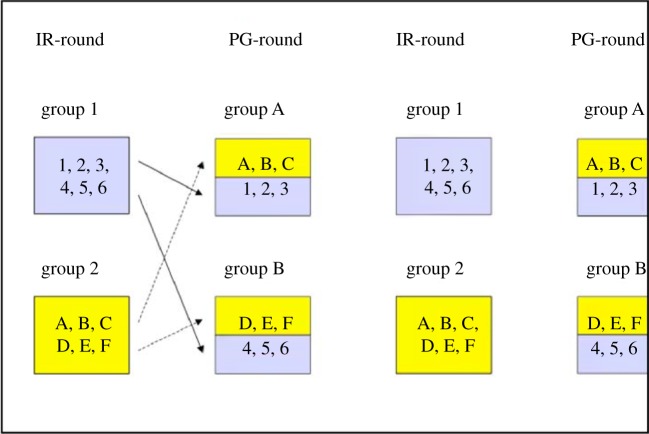
Group composition in public goods (PG) and indirect reciprocity (IR) rounds in each of six IR-rounds and in each of six PG-rounds. The cut-off of the figure in the second PG-round indicates that this sequence is continued. See §8 for details.

To test this prediction, Semmann *et al.* [40] had groups of 12 students each playing simultaneously. They were informed that six of them would play together in one group for the pair (i.e. indirect reciprocity) game and the other six in another group for the same type of game (see figure 5 for the experimental design). The same six people would play the pair game repeatedly and they could recognize their co-players by their pseudonyms. Every second round they would play the group (i.e. public goods) game. For this game, three people of each group for the pair game would be combined with three people of the other pair game group. Also this group composition would remain stable whenever they played the group game. Again they would recognize their co-players by their pseudonyms. Everybody could observe every interaction on the screen and the sequence of ‘yes’ and ‘no’ decisions of each potential receiver was displayed in the indirect reciprocity rounds. There were six rounds of each type of game. Tests ensured that the subjects had understood the design. Now we can compare how the same subject reacted to a ‘no’ (or ‘yes’) given in the previous public goods round in the indirect reciprocity round when the receiver had been either in the donor's own or in the other public goods group. Surprisingly, the subjects did not treat own group members and foreign group members differently, even though they recognized them. This suggests that reputation is a label that is taken into account in social interactions irrespective of where this reputation has been gained. The label says ‘this is a helpful person deserving to be trusted’.

## 9. Gaining good reputation through donations to charity

If reputation is transferable between social groups transporting the message that a person is a valuable and trustworthy social partner, the same kind of reliable reputation could be gained by giving to charity. It has to be ensured, however, that people know about it. Milinski *et al.* [37] did an experiment in which groups of six subjects each played several rounds of the indirect reciprocity game. After each episode of being asked: ‘do you want to give DM (= Deutschemark, before € in Germany) 2.50 to this person?’ (if yes, the receiver obtained DM 4), the player was asked: ‘do you want to give DM 2.50 to UNICEF?’ (if yes, UNICEF obtained DM 4). It was made clear that the amount of money on UNICEF's account would be sent to UNICEF and every player would receive a copy of the receipt. As a result, giving to UNICEF was rewarded in the next round of indirect reciprocity; not giving was not rewarded.

Even a reputation gained through giving money away from the group is rewarded in indirect reciprocity rounds. However, this worked only when donations to UNICEF were done ‘in public’, i.e. every group member could see it with the donor's pseudonym. In control groups in which the subjects knew that no information about their donations would be announced to the group, dramatically less money was donated privately to UNICEF. Again cooperation was conditional on others being able to see it. For further examples of how making contributions in public helps cooperation see [83,84].

## 10. Gaining reputation through averting climate change

The Intergovernmental Panel on Climate Change (IPCC) published its fourth report early in 2007, saying that to reduce the risk of dangerous climate change, global greenhouse gas emissions should be reduced to about 50% of the present level by 2050. While substantial emission reductions are likely to have negative short-term economic effects, failure to accomplish this reduction may well incur dangerous climate change later, resulting in substantial human, ecological and economic losses. Preserving the global climate is a natural public goods game. All people on Earth take part in this game. Milinski *et al.* [85] asked groups of six students each to invest in a ‘climate public goods’ pool, either with their pseudonym, recognizable in the alternating indirect reciprocity game, or they were completely anonymous. The money in the climate public goods pool was, after doubling, not distributed among the players but used for an advertisement in a widely read daily newspaper, with information about the global climate and simple rules for reducing CO_2_ emission. Every second group received written expert information about the state of climate research from the Max Planck Institute for Meteorology.

**Figure 6.**
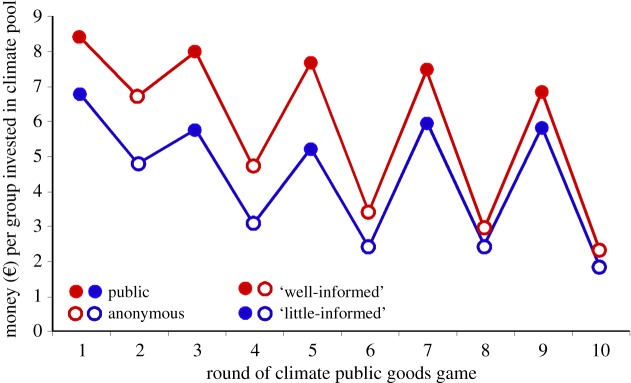
Money (€) per group of six subjects invested in a climate pool in each round of the non-anonymous (filled) and anonymous (open) climate public goods game. In one treatment (well-informed), the groups received additional expert information about the state of the global climate (red); in the other treatment (little-informed), the groups received no additional information (blue) (adapted from [85]).

The players invested more in the climate account when they were provided with expert information (figure 6). Personal investments in climate protection, however, increased substantially if players could invest publicly, thus gaining social reputation. This increase occurred because subjects rewarded other subjects' contributions to sustaining the climate, thus reinforcing their altruism. Therefore, altruism may convert to net personal benefit and to relaxing the dilemma if the gain in reputation is large enough. The finding that people reward contributions to sustaining the climate is a surprising result. There are obvious ways these unexpected findings can be applied on a large scale.

## 11. The efficient interaction of reputation building and costly punishment

**Figure 7.**
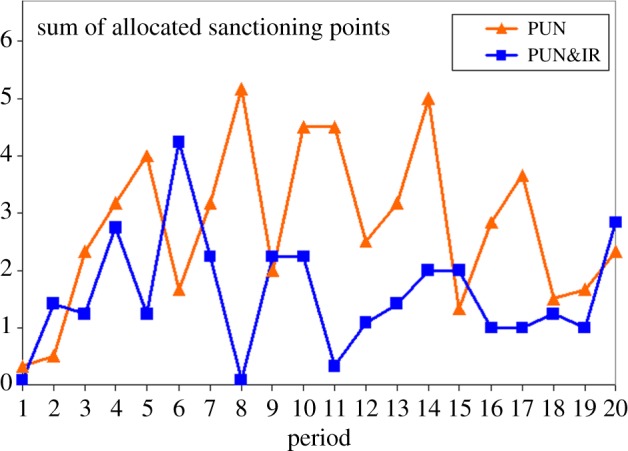
Sum of allocated sanctioning points in the treatments ‘punishment, PUN’ and ‘punishment and indirect reciprocity, PUN&IR’ in each of 20 periods (adapted from [86]).

‘Disciplining’ non-cooperators can be achieved through punishment and reputation building. Punishment incurs salient costs for both the punisher and the punished, whereas reputation mechanisms discipline by withholding action, saving costs for the ‘punisher’. As a consequence, costly punishment may become extinct in environments in which effective reputation building—for example, through indirect reciprocity—provides a cheaper and powerful way to maintain cooperation. Unexpectedly, however, punishment is maintained at a low level when a combination with reputation building is available [86]. Costly punishment acts are markedly reduced although not simply substituted by appreciating reputation. Indeed, the remaining punishment acts are concentrated on free-riders, who are most severely punished in the combination. When given a choice, subjects even prefer a combination of reputation building with costly punishment. The interaction between punishment and reputation building boosts cooperative efficiency (figure 7). Because punishment and reputation building are omnipresent interacting forces in human societies, costly punishing should appear less destructive without losing its deterring force. Reputation is not only a currency in social interactions, it also helps to change our world to be less punchy and more diplomatic.

## 12. Conclusion

I have concentrated on the role of reputation when individuals expect help from someone whom they did not help themselves. This ‘stranger’ has an incentive to help if the individual in need of help has previously helped others (indirect reciprocity). The stranger might have observed these actions or heard gossip about them. Evolutionary theory predicts that if the person has helped more often than refused help they have positive image score or reputation and should be helped [33]. For indirect reciprocity, reputation needs to be known; for direct reciprocation of help remembering a face is enough. Any costly investment in others increases a person's reputation. Reputation is a strong driver of cooperation, serving as a currency for future social exchange [87]. The finding that pictures of watching eyes enhance both cooperation and pro-social behaviour of young children suggests evolved (gene programmed) cooperation.

## References

[1] Trivers RL 1971 The evolution of reciprocal altruism. Q. Rev. Biol. 46, 35–57. (10.1086/406755)

[2] Berg J, Dickhaut J, McCabe K 1995 Trust, reciprocity, and social-history. Games Econ. Behav. 10, 122–142. (10.1006/game.1995.1027)

[3] Duchenne de Boulogne G 1862 The mechanism of human face expression. Paris, France: Jules Renard.

[4] Centorrino S, Djemai E, Hopfensitz A, Milinski M, Seabright P 2015 A model of smiling as costly signal of cooperation opportunities. Adapt. Hum. Behav. Physiol. 1, 2–18. (10.1007/s40750-015-0026-4)

[5] Centorrino S, Djemai E, Hopfensitz A, Milinski M, Seabright P 2015 Honest signalling in trust interactions: smiles rated as genuine induce trust and signal higher earning opportunities. Evol. Hum. Behav. 36, 8–16. (10.1016/j.evolhumbehav.2014.08.001)

[6] McGregor PK 1993 Signalling in territorial systems: a context for individual identification, ranging and eavesdropping. Phil. Trans. R. Soc. Lond. *B* 340, 237–244. (10.1098/rstb.1993.0063)

[7] Earley RL 2010 Social eavesdropping and the evolution of conditional cooperation and cheating strategies. Phil. Trans. R. Soc. B 365, 2675–2686. (10.1098/rstb.2010.0147) PMC2936174 20679111

[8] Rockenbach B, Milinski M 2011 To qualify as a social partner, humans hide severe punishment, although their observed cooperativeness is decisive. Proc. Natl Acad. Sci. USA 108, 18 307–18 312. (10.1073/pnas.1108996108) 21987800 PMC3215000

[9] Akçay Ç, Reed VA, Campbell SE, Templeton CN, Beecher MD 2010 Indirect reciprocity: songsparrows distrust aggressive neighbours based on eavesdropping. Anim. Behav. 80, 1041–1047. (10.1016/j.anbehav.2010.09.009)

[10] Ohtsuki H, Nowak MA 2009 Indirect reciprocity provides only a narrow margin of efficiency for costly punishment. Nature 457, 79–82. (10.1038/nature07601) 19122640 PMC2614697

[11] Sommerfeld R, Krambeck H-J, Milinski M 2008 Multiple gossip statements and their effect on reputation and trustworthiness. Proc. R. Soc. R. B 275, 2529–2536. (10.1098/rspb.2008.0762) PMC2603203 18664435

[12] Ostrom E 1998 A behavioural approach to the rational choice theory of collective action. Am. Polit. Sci. Rev. 92, 1–22. (10.2307/2585925)

[13] Trivers R 1985 Social evolution. Menlo Park, CA: Benjamin/Cummings.

[14] McElreath R et al. 2002 The role of cognition and emotion in cooperation. In Genetic and cultural evolution of cooperation (ed. Hammerstein P), pp. 125–152. Cambridge, MA: MIT Press.

[15] Pounstone W 1992 Prisoner's Dilemma. New York, NY: Oxford University Press.

[16] Axelrod R 1984 The evolution of cooperation. New York, NY: Basic Books.

[17] Axelrod R, Hamilton WD 1981 The evolution of cooperation. Science 211, 1390–1396. (10.1126/science.7466396) 7466396

[18] Wilkinson GS 1984 Reciprocal food sharing in the vampire bat. Nature 308, 181–184. (10.1038/308181a0)

[19] Milinski M 1987 TIT FOR TAT in sticklebacks and the evolution of cooperation. Nature 325, 433–435. (10.1038/325433a0) 3808044

[20] Rutte C, Taborsky M 2008 The influence of social experience on cooperative behaviour of rats (*Rattus norvegicus*): direct vs generalised reciprocity. Behav. Ecol. Sociobiol. 62, 499–505. (10.1007/s00265-007-0474-3)

[21] Krams I, Krama T, Igaune K 2006 Mobbing behaviour: reciprocity-based co-operation in breeding pied flycatchers *Ficedula hypoleuca*. Ibis 148, 50–54. (10.1111/j.1474-919X.2006.00480.x)

[22] Gomes CM, Boesch C 2011 Reciprocity and trades in wild West African chimpanzees. Behav. Ecol. Sociobiol. 65, 2183–2196. (10.1007/s00265-011-1227-x)

[23] Milinski M, Wedekind C 1998 Working memory constrains human cooperation in the Prisoner's Dilemma. Proc. Natl Acad. Sci. USA 95, 13 755–13 758. (10.1073/pnas.95.23.13755) 9811873 PMC24892

[24] Nowak M, Sigmund K 1992 Tit for tat in heterogeneous populations. Nature 355, 250–253. (10.1038/355250a0)

[25] Nowak M, Sigmund K 1993 A strategy of win-stay, lose-shift that outperformes tit-for-tat in the Prisoner's Dilemma game. Nature 364, 56–58. (10.1038/364056a0) 8316296

[26] Milinski M 1993 Cooperation wins and stays. Nature 364, 12–13. (10.1038/364012a0) 8316291

[27] Press WH, Dyson FJ 2012 Iterated Prisoner's Dilemma contains strategies that dominate any evolutionary opponent. Proc. Natl Acad. Sci. USA 109, 10 409–10 413. (10.1073/pnas.1206569109) PMC3387070 22615375

[28] Hilbe C, Roehl T, Milinski M 2014 Extortion subdues human players but is finally punished in the prisoner's dilemma. Nat. Commun. 5, 3976 (10.1038/ncomms4976) 24874294 PMC4050275

[29] Feinberg M, Willer R, Schultz M 2014 Gossip and ostracism promote cooperation in groups. Psychol. Sci. 25, 656–664. (10.1177/0956797613510184) 24463551

[30] Alexander RD 1987 The biology of moral systems. New York, NY: De Gruyter.

[31] Zahavi A 1991 On the definition of sexual selection, Fisher's model, and the evolution of waste and of signals in general. Anim. Behav. 42, 501–503. (10.1016/S0003-3472(05)80052-1)

[32] Nowak MA, Sigmund K 1998 The dynamics of indirect reciprocity. J. Theor. Biol. 194, 561–574. (10.1006/jtbi.1998.0775) 9790830

[33] Nowak MA, Sigmund K 1998 Evolution of indirect reciprocity by image scoring. Nature 393, 573–577. (10.1038/31225) 9634232

[34] Wedekind C, Milinski M 2000 Cooperation through image scoring in humans. Science 288, 850–852. (10.1126/science.288.5467.850) 10797005

[35] Bolton GE, Katok E, Ockenfels A 2005 Cooperation among strangers with limited information about reputation. J. Public Econ. 89, 1457–1468. (10.1016/j.jpubeco.2004.03.008)

[36] Milinski M, Semmann D, Bakker TCM, Krambeck H-J 2001 Cooperation through indirect reciprocity: image scoring or standing strategy? Proc. R. Soc. Lond. B 268, 2495–2501. (10.1098/rspb.2001.1809) PMC1088906 11747570

[37] Milinski M, Semmann D, Krambeck HJ 2002 Donors to charity gain in both indirect reciprocity and political reputation. Proc. R. Soc. Lond. B 269, 881–883. (10.1098/rspb.2002.1964) PMC1690974 12028769

[38] Seinen I, Schram A 2006 Social status and group norms: indirect reciprocity in a repeated helping experiment. Eur. Econ. Rev. 50, 581–602. (10.1016/j.euroecorev.2004.10.005)

[39] Semmann D, Krambeck HJ, Milinski M 2004 Strategic investment in reputation. Behav. Ecol. Sociobiol. 56, 248–252. (10.1007/s00265-004-0782-9)

[40] Semmann D, Krambeck HJ, Milinski M 2005 Reputation is valuable within and outside one's own social group. Behav. Ecol. Sociobiol. 57, 611–616. (10.1007/s00265-004-0885-3)

[41] Pfeiffer T, Rutte C, Killingback T, Taborsky M, Bonhoeffer S 2005 Evolution of cooperation by generalized reciprocity. Proc. R. Soc. B 272, 1115–1120. (10.1098/rspb.2004.2988) PMC1559812 16024372

[42] Boyd R, Richerson P 1989 The evolution of indirect reciprocity. Soc. Netw. 11, 213–236. (10.1016/0378-8733(89)90003-8)

[43] Nowak MA, Roch S 2007 Upstream reciprocity and the evolution of gratitude. Proc. R. Soc. B 274, 605–610. (10.1098/rspb.2006.0125) PMC2197219 17254983

[44] Roberts G 1998 Competitive altruism: from reciprocity to the handicap principle. Proc. R. Soc. Lond. B 265, 427–431. (10.1098/rspb.1998.0312)

[45] Barclay P, Willer R 2007 Partner choice creates competitive altruism in humans. Proc. R. Soc. B 274, 749–753. (10.1098/rspb.2006.0209) PMC2197220 17255001

[46] Leimar O, Hammerstein P 2001 Evolution of cooperation through indirect reciprocity. Proc. R. Soc. Lond. B 268, 745–753. (10.1098/rspb.2000.1573) PMC1088665 11321064

[47] Sugden R 1986 The economics of rights, co-operation and welfare. Oxford, UK: Basil Blackwell.

[48] Ohtsuki H, Iwasa Y 2006 The leading eight: social norms that can maintain cooperation by indirect reciprocity. J. Theor. Biol. 239, 435–444. (10.1016/j.jtbi.2005.08.008) 16174521

[49] Nax HH, Perc M, Szolnoki A, Helbing D 2015 Stability of cooperation under image scoring in group interactions. Sci. Rep. 5, 1–7. (10.9734/JSRR/2015/14076) PMC4502532 26177466

[50] Berger U, Grüne A. 2014 Evolutionary stability of indirect reciprocity by image scoring. Working paper no. 168, pp. 1–22. Department of Economics, Vienna University of Economics and Business.

[51] McNamara JM, Doodson P 2015 Reputation can enhance or suppress cooperation through positive feedback. Nat. Commun. 6, 1–7. (10.1038/ncomms7134) 25601004

[52] Hardin G 1968 Tragedy of commons. Science 162, 1243–1248. (10.1126/science.162.3859.1243) 5699198

[53] Hardin G 1998 Extensions of ‘the tragedy of the commons’. Science 280, 682–683. (10.1126/science.280.5364.682)

[54] IPCC. 2013 Summary for policymakers. In Climate Change 2013: The physical science basis. Contribution of Working Group I to the Fifth Assessment Report of the Intergovernmental Panel on Climate Change (eds Stocker TF et al.), pp. 3–29. New York, NY: Cambridge University Press.

[55] Ledyard JO 1995 Handbook of experimental economics (eds Kagel JH, Roth AE), pp. 111–194. Princeton, NJ: Princeton University Press.

[56] Yamagishi T 1986 The provision of a sanctioning system as a public good. J. Pers. Soc. Psychol. 51, 110–116. (10.1037/0022-3514.51.1.110)

[57] Boyd R, Richerson PJ 1992 Punishment allows the evolution of cooperation (or anything else) in sizable groups. Ethol. Sociobiol. 13, 171–195. (10.1016/0162-3095(92)90032-Y)

[58] Fehr E, Gächter S 2002 Altruistic punishment in humans. Nature 415, 137–140. (10.1038/415137a) 11805825

[59] Traulsen A, Röhl T, Milinski M 2012 An economic experiment reveals that humans prefer pool punishment to maintain the commons. Proc. R. Soc. B 279, 3716–3721. (10.1098/rspb.2012.0937) PMC3415907 22764167

[60] Dreber A, Rand DG, Fudenberg D, Nowak MA 2008 Winners don't punish. Nature 452, 348–351. (10.1038/nature06723) 18354481 PMC2292414

[61] Gächter S, Renner E, Sefton M 2008 The long-run benefits of punishment. Science 322, 1510–1510. (10.1126/science.1164744) 19056978

[62] Gürerk O, Irlenbusch B, Rockenbach B 2006 The competitive advantage of sanctioning institutions. Science 312, 108–111. (10.1126/science.1123633) 16601192

[63] Sutter M, Haigner S, Kocher M 2010 Choosing the carrot or the stick? Endogenous institutional choice in social dilemma situations. Rev. Econ. Stud. 77, 1540–1566. (10.1111/j.1467-937X.2010.00608.x)

[64] Sefton M, Shupp R, Walker J 2007 The effect of rewards and sanctions in provision of public goods. Econ. Inquiry 45, 671–690. (10.1111/j.1465-7295.2007.00051.x)

[65] Dickinson D 2001 The carrot vs. the stick in work team motivation. Exp. Econ. 4, 107–124. (10.1023/A:1011449606931)

[66] Andreoni J, Harbough W, Versterlund L 2003 The carrot or the stick: rewards, punishments, and cooperation. Am. Econ. Rev. 93, 893–902. (10.1257/000282803322157142)

[67] Milinski M, Rockenbach B 2012 On the interaction of the stick and the carrot in social dilemmas. J. Theor. Biol. 299, 139–143. (10.1016/j.jtbi.2011.03.014) 21458464

[68] Raihani NJ, Bshary R 2015 The reputation of punishers. Trends Ecol. Evol. 30, 98–103. (10.1016/j.tree.2014.12.003) 25577128

[69] Milinski M, Semmann D, Krambeck HJ 2002 Reputation helps solve the 'tragedy of the commons’. Nature 415, 424–426. (10.1038/415424a) 11807552

[70] Panchanathan K, Boyd R 2004 Indirect reciprocity can stabilize cooperation without the second-order free rider problem. Nature 432, 499–502. (10.1038/nature02978) 15565153

[71] Barclay P 2004 Trustworthiness and competitive altruism can also solve the ‘tragedy of the commons’. Evol. Hum. Behav. 25, 209–220. (10.1016/j.evolhumbehav.2004.04.002)

[72] Fehr E 2004 Don't lose your reputation. Nature 432, 449–450. (10.1038/432449a) 15565133

[73] Pollock G, Dugatkin LA 1992 Reciprocity and the emergence of reputation. J. Theor. Biol. 159, 25–37. (10.1016/S0022-5193(05)80765-9)

[74] Ostrom E 2003 Toward a behavioral theory linking trust, reciprocity, and reputation. In Trust and reciprocity: interdisciplinary lessons from experimental research, vol. 6 (eds Ostrom E, Walker J), pp. 19–79. Russell Sage Foundation Series on Trust New York, NY: Russell Sage Foundation.

[75] Bshary R 2002 Biting cleaner fish use altruism to deceive image-scoring client reef fish. Proc. R. Soc. Lond. B 269, 2087–2093. (10.1098/rspb.2002.2084) PMC1691138 12396482

[76] Haley KJ, Fessler DMT 2005 Nobody's watching? Subtle cues affect generosity in an anonymous economic game. Evol. Hum. Behav. 26, 245–256. (10.1016/j.evolhumbehav.2005.01.002)

[77] Bateson M, Nettle D, Roberts G 2006 Cues of being watched enhance cooperation in a real-world setting. Biol. Lett. 2, 412–414. (10.1098/rsbl.2006.0509) 17148417 PMC1686213

[78] Nettle D, Harper Z, Kidson A, Stone R, Penton-Voak IS, Bateson M 2013 The watching eyes effect in the Dictator Game: it's not how much you give, it's being seen to give something. Evol. Hum. Behav. 34, 35–40. (10.1016/j.evolhumbehav.2012.08.004)

[79] Bentin S, Allison T, Puce A, Perez E, McCarthy G 1996 Electrophysiological studies of face perception in humans. J. Cogn. Neurosci. 8, 551–565. (10.1162/jocn.1996.8.6.551) 20740065 PMC2927138

[80] Emery NJ 2000 The eyes have it: the neuroethology, function and evolution of social gaze. Neurosci. Biobehav. Rev. 24, 581–604. (10.1016/S0149-7634(00)00025-7) 10940436

[81] Milinski M, Rockenbach B 2007 Spying on others evolves. Science 317, 464–465. (10.1126/science.1143918) 17656712

[82] Blake PR, McAuliffe K 2011 ‘I had so much it didn't seem fair’: eight-year-olds reject two forms of inequity. Cognition 120, 215–224. (10.1016/j.cognition.2011.04.006) 21616483

[83] Alpizar F, Carlsson F, Johansson-Stenman O 2008 Anonymity, reciprocity, and conformity: evidence from voluntary contributions to a national park in Costa Rica. J. Public Econ. 92, 1047–1060. (10.1016/j.jpubeco.2007.11.004)

[84] Yoeli E, Hoffman M, Rand DG, Nowak MA 2013 Powering up with indirect reciprocity in a large-scale field experiment. Proc. Natl Acad. Sci. USA 110(Suppl 2), 10 424–10 429. (10.1073/pnas.1301210110) PMC3690615 23754399

[85] Milinski M, Semmann D, Krambeck HJ, Marotzke J 2006 Stabilizing the Earth's climate is not a losing game: supporting evidence from public goods experiments. Proc. Natl Acad. Sci. USA 103, 3994–3998. (10.1073/pnas.0504902103) 16537474 PMC1449634

[86] Rockenbach B, Milinski M 2006 The efficient interaction of indirect reciprocity and costly punishment. Nature 444, 718–723. (10.1038/nature05229) 17151660

[87] Milinski M 2016 Reputational commons. In The SAGE Encyclopedia of corporate reputation. (ed. Carroll CE). Thousand Oaks, CA: Sage.

